# Safety of Acupuncture in Patients Taking Newer Oral Anticoagulants: A Retrospective Chart Review Study

**DOI:** 10.1155/2018/8042198

**Published:** 2018-10-10

**Authors:** Seungwon Kwon, Woo-Sang Jung, Seungbo Yang, Chul Jin, Seung-Yeon Cho, Seong-Uk Park, Sang-Kwan Moon, Jung-Mi Park, Chang-Nam Ko, Ki-Ho Cho, Min-Jeong Park

**Affiliations:** Department of Cardiology and Neurology, College of Korean Medicine, Kyung Hee University, Seoul, Republic of Korea

## Abstract

Anticoagulant therapy is used to reduce the risk of thromboembolic events in patients with atrial fibrillation. Warfarin has been the traditional anticoagulant but is difficult to use because of its narrow therapeutic window. Recently, newer oral anticoagulants (NOACs) have been developed. However, bleeding continues to be a significant complication. The objective of this study was to assess the safety of acupuncture in patients taking NOACs. The medical records in the Stroke Center at Kyung Hee University Korean Medicine Hospital were retrospectively reviewed to identify patients who had received acupuncture between January 2017 and September 2017. The patients were divided into groups according to whether they were taking an NOAC, an antiplatelet agent, or no anticoagulant therapy. Bleeding-related side effects that occurred immediately after removal of acupuncture needles were recorded. Three hundred and sixteen patients underwent 10,177 acupuncture sessions during the study period. Microbleeding (bleeding that ceased within 30 s) occurred at a rate of 3.9% in the NOAC group, 5.6% in the antiplatelet group, and 5.1% in the control group. There were no between-group differences in the microbleeding rate. No serious adverse events, including major bleeding, were detected. These findings indicate that acupuncture is safe in patients taking NOACs.

## 1. Introduction

Newer oral anticoagulants (NOACs) have been developed to overcome the limitations of warfarin, a conventional vitamin K antagonist that has been used widely to reduce the risk of cardiovascular events in patients with atrial fibrillation [1–3]. The NOACs include dabigatran, a direct thrombin inhibitor, and rivaroxaban, apixaban, and edoxaban, all of which are direct coagulation factor Xa inhibitors. These anticoagulants have been confirmed to be as effective as warfarin [4–7] and to have predictable pharmacokinetics, so regular monitoring of blood levels is not necessary when using these agents. Furthermore, they have a rapid onset of action and reversal of effect soon after cessation, as well as limited drug and food interactions. Therefore, NOACs can be expected to replace warfarin in patients requiring anticoagulation therapy.

However, there is concern about the risk of bleeding associated with long-term use of anticoagulants. Observational studies in patients receiving warfarin have reported that fatal bleeding-related side effects occur at a rate of 0.6%, intracerebral or intraperitoneal hemorrhage at a rate of 3.0%, and severe microbleeding at a rate of 9.6% [8]. The risk of bleeding events in patients receiving NOACs has been reported to be similar to or lower than that in those receiving warfarin [9].

Several studies have investigated the dose-dependent safety of acupuncture [10–16]. Several complications have been reported, but fatal side effects are extremely rare and generally have occurred when acupuncture has been administered by inexperienced operators. A systematic review of the safety of acupuncture in 384 patients receiving an anticoagulant (warfarin, urokinase, or heparin) revealed that microhemorrhage occurred in 56 (1.4%) of 3974 patients who received acupuncture and that only one patient (0.02%) experienced organ damage because of inappropriately deep needling. These findings indicated that acupuncture is safe for patients receiving anticoagulation therapy [17].

Although the safety of acupuncture in patients receiving anticoagulation therapy has been investigated in previous studies, there has been no report on patients taking NOACs. In this study, we compared the safety of acupuncture in patients who were receiving an NOAC with that in patients who were receiving conventional or no anticoagulation.

## 2. Materials and Methods

### 2.1. Study Design

All patients admitted to the Stroke Center, Kyung Hee University Korean Medicine Hospital, receive acupuncture once or twice daily. The acupuncture is performed routinely by Korean doctors who are graduates of a school of Korean medicine. Disposable needles (Dongbang Acupuncture Inc., Boryeong, Korea) measuring 0.25 mm × 40 mm or 0.25 mm × 30 mm are inserted into each acupuncture point to a depth of 10–20 mm, depending on the point selected, for approximately 20 min. Immediately upon withdrawal of the needle, any side effects at the acupuncture site are recorded on a routine checklist by the medical staff (Table 1). The doctors knew what medication the patient was taking.

We searched the medical records of patients who were hospitalized between January 2017 and September 2017 and received acupuncture. The patients were divided into groups according to whether they were taking an NOAC (group A), an antiplatelet agent (group B), no NOAC or antiplatelet agent (group C [control]), or warfarin (group D) at the time of acupuncture. There were no restrictions with regard to sex, age, diagnosis, severity of symptoms, hospital stay, or duration or type of therapy. Patients whose medical records did not mention side effects of acupuncture were excluded. Patients who experienced sudden bleeding unrelated to acupuncture, such as trauma or gastrointestinal bleeding, were also excluded, as were patients with an activated partial thromboplastin time >45 sec, a prothrombin time/international normalized ratio >3.0, a prothrombin time >15 sec, or a platelet count <100,000/mm^3^ on admission.

### 2.2. Outcome Assessments

The safety of acupuncture was assessed using a checklist (Table 1). Multiple needle insertions in the same patient during one procedure were considered a single session.

### 2.3. Ethical Approval

The study protocol was approved by the Institutional Review Board of Kyung Hee University Korean Medicine Hospital (KOMCIRB-170915-HR-038) and performed in accordance with the ethical standards set down in the Declaration of Helsinki.

### 2.4. Statistical Analysis

The data were compared between the groups using the Statistical Package for Social Sciences for Windows version 18.0 (IBM Corp., Armonk, NY, USA). Continuous variables, including age and side effects, were compared between the groups using one-way analysis of variance and categorical variables were compared using the chi-squared test. A* p* value < 0.05 was considered statistically significant.

## 3. Results

Review of the medical records identified 324 patients who had received acupuncture at the Stroke Center, Kyung Hee University Korean Medicine Hospital, between January 2017 and September 2017. Only six patients were receiving warfarin, so they were excluded from the statistical analysis, leaving 316 patients who were assigned to group A (n = 28), group B (n = 145), or group C (n = 143).

### 3.1. Baseline Characteristics

There was no statistically significant difference in the sex distribution between the groups. However, there were significant differences in patient age (*p* < 0.001) and the numbers of patients with atrial fibrillation (*p* < 0.001), hypertension (*p* = 0.005), and diabetes mellitus (*p*=0.048) between the groups. The NOAC agents taken in group A were apixaban, dabigatran, and rivaroxaban. The antiplatelet agents taken in group B, in descending order of frequency, were aspirin as monotherapy, a combination of aspirin and clopidogrel, clopidogrel, or triflusal as monotherapy, and a combination of aspirin and triflusal. Two patients in group A were taking two antiplatelet agents concomitantly (apixaban and aspirin in one case and dabigatran and clopidogrel in the other; Table 2).

### 3.2. Side Effects of Acupuncture

In total, 1076 acupuncture sessions were evaluated in group A, 4574 in group B, and 4527 in group C (Table 3). The most common side effect was microbleeding; the incidence was not significantly different between the study groups (*p* = 0.084) and followed a descending order of group B, group C, and group A. Microbleeding was defined as bleeding that took from 10 s to 30 s to resolve. Most episodes of microbleeding stopped within 10 s to 20 s. There was no significant difference in the rate of cessation of microbleeding in 10–20 s between the two groups (*p* = 0.065). There were no serious adverse events, including no instances of massive hemorrhage.

## 4. Discussion

The purpose of this study was to evaluate the safety of acupuncture in patients receiving NOACs after admission to the Stroke Center, Kyung Hee University Korean Medicine Hospital. The side effects evaluated in the study were microbleeding, edema, dizziness, fatigue, nausea and vomiting, pneumothorax, needle fracture, skin rash, and pain.

The total incidence of side effects was 4.0% in group A, 6.9% in group B, and 5.5% in group C. There were no serious side effects, such as excessive bleeding or pneumothorax. The incidence of side effects was significantly lower in group A than in group B or group C. However, the incidence of adverse events in group A is not low compared to previous reports of acupuncture adverse event [17]. In addition, the incidence of most of the side effects, microbleeding, was not significantly different between the three groups. Therefore, we interpret group A as having similar risks as group B and group C.

As in a number of previous studies [10–16], there were no fatalities after acupuncture in the present study. The most common side effect was microbleeding that took less than 30 s to resolve after needle removal. The incidence of microbleeding was 3.9% in group A, 5.6% in group B, and 5.1% in group C; the differences were not statistically significant (*p* = 0.084). In previous studies, the incidence of microbleeding was reported to be 8.4% in patients treated with acupuncture [16], so the incidence in all our study groups was lower than that in previous reports.

Kim et al [18] reported that the incidence of microbleeding after acupuncture was 4.8% in their warfarin-treated group, 0.9% in their antiplatelet-treated group, and 3.0% in their untreated group. The incidence of microbleeding was lower in the NOAC group than in the warfarin group when compared with the incidence in this study.

The incidence of microbleeding was 3.9% in the patients who received NOAC and occurred between 10 and 20 s after needle removal. The only other side effect reported was bruising, which occurred in one patient. Therefore, acupuncture administered by a specialist is safe and need not be contraindicated in patients taking an NOAC.

The main limitation of this study is its small sample size. The number of patients in the NOAC group was particularly small and the number of patients taking warfarin was so small that no comparisons involving warfarin could be made. Also, this study is a retrospective study. Practitioners already knew that patients were taking an NOAC. There is a possibility that this has shown carefulness in the procedure. Large-scale, well-designed, double-blind, prospective studies are needed to overcome these limitations. However, there were no significant side effects after acupuncture in the patients taking an NOAC, and there was no significant difference in the incidence of side effects between patients taking antiplatelet therapy and the controls. These results support the view that acupuncture is relatively safe in patients taking an NOAC.

## 5. Conclusions

These findings indicate that acupuncture is safe in patients taking NOACs. Further large and rigorous clinical trials are needed.

## Figures and Tables

**Table 1:** Checklist for acupuncture-related side effects.

**Item**	**Definition**
**Microbleeding**	The number of microbleeds was checked according to the time of hemostasis 10 s after removal of the acupuncture needle. This procedure was routinely carried out by Korean medicine doctors. Bleeding that stopped within 30 s was defined as “microbleeding.” Multiple bleeding events occurring during one acupuncture session were considered to be one episode of bleeding.
**Extensive bleeding**	Bleeding that took at least 30 s to stop.
**Time taken to achieve hemostasis**	
**Massive hemorrhage influencing vital signs or causing other complications**	
**Bruising**	Bruising checked 1–3 h after removing the needles.
**Edema**	
**Faintness or dizziness, fatigue or exhaustion, nausea or vomiting**	
**Pneumothorax**	
**Needle fracture**	
**Skin eruption or itching**	
**Pain after needling**	
**Other adverse effects**	

**Table 2:** General patient characteristics.

	Group A (n = 28)	Group B (n = 145)	Group C (n = 143)	*p* value^a^
**Sex**, n (%)				
Male	14 (50.0)	78 (53.8)	64 (44.8)	0.308
Female	14 (50.0)	67 (46.2)	79 (55.2)	
**Age, years**	73.14 ± 9.60	67.52 ± 11.44	62.60 ± 13.42	<0.001
**Medical history, n (**%**)**				
Atrial fibrillation	24 (85.7)	1 (0.7)	4 (2.7)	<0.001
Hypertension	13 (41.9)	96 (66.2)	72 (50.3)	0.005
Diabetes mellitus	8 (22.2)	58 (28.2)	31 (17.8)	0.048
Hyperlipidemia	5 (15.2)	53 (26.8)	31 (17.8)	0.071
**NOAC therapy, n (**%**)**				
Dabigatran	11 (39.3)	0	0	
Rivaroxaban	2 (7.1)	0	0	
Apixaban	15 (53.6)	0	0	
**Antiplatelet therapy**, n (%)				
Aspirin	1	50 (34.5)	0	
Clopidogrel	1	35 (24.1)	0	
Triflusal	0	7 (4.8)	0	
Aspirin + clopidogrel	0	46 (31.7)	0	
Aspirin + triflusal	0	7 (4.8)	0	

The data are shown as the mean ± standard deviation or number (percentage). Group A includes patients taking an NOAC. Group B includes patients taking an antiplatelet agent. Group C includes the controls who were not taking an NOAC or antiplatelet agent. ^a^Analysis of variance used for age and the chi-squared test for categorical variables.

**Table 3:** Side effects of acupuncture in the study groups.

	Group A	Group B	Group C	*p* value^a^
	(n = 28)	(n = 145)	(n = 143)	
Total number of acupuncture treatments, n	1076	4574	4527	
Total number of side effects	43 (4.0)	314 (6.9)	250 (5.5)	<0.001
Microbleeding (10 s ≤ x < 30 s)	42 (3.9)	254 (5.6)	230 (5.1)	0.084
10 s ≤ x < 20 s	42 (3.8)	217 (4.5)	203 (4.3)	0.065
20 s ≤ x < 30 s	0 (0.0)	37 (0.8)	27 (0.6)	
Extensive bleeding	0 (0.0)	4 (0.0)	2 (0.0)	
Massive hemorrhage	0 (0.0)	0 (0.0)	0 (0.0)	
Bruising	1 (0.0)	31 (0.6)	13 (0.2)	
Edema	0 (0.0)	22 (0.4)	4 (0.0)	
Faintness or dizziness	0 (0.0)	0 (0.0)	1 (0.0)	
Fatigue or exhaustion	0 (0.0)	0 (0.0)	0 (0.0)	
Nausea or vomiting	0 (0.0)	0 (0.0)	0 (0.0)	
Pneumothorax	0 (0.0)	0 (0.0)	0 (0.0)	
Needle fracture	0 (0.0)	0 (0.0)	0 (0.0)	
Skin eruption or itching	0 (0.0)	0 (0.0)	0 (0.0)	
Pain after needling	0 (0.0)	3 (0.0)	0 (0.0)	
Other adverse effects	0 (0.0)	0 (0.0)	0 (0.0)	

The data are shown as the number (percentage). Group A includes patients taking an NOAC. Group B includes patients taking an antiplatelet agent. Group C includes the controls who were not taking an NOAC or antiplatelet agent. ^a^Chi-squared test.

## Data Availability

The data used to support the findings of this study is not available, because it is medical records.

## References

[1] Go A. S., Hylek E. M., Phillips K. A. (2001). Prevalence of diagnosed atrial fibrillation in adults: National implications for rhythm management and stroke prevention: The anticoagulation and risk factors in atrial fibrillation (ATRIA) study. *Journal of the American Medical Association*.

[2] Lloyd-Jones D., Adams R. J., Brown T. M. (2010). Executive summary: heart disease and stroke statistics-2010 update: a report from the American Heart Association. *Circulation*.

[3] Ezekowitz M. D., Bridgers S. L., James K. E. (1992). Warfarin in the Prevention of Stroke Associated with Nonrheumatic Atrial Fibrillation. *The New England Journal of Medicine*.

[4] Patel M. R., Mahaffey K. W., Garg J. (2011). Rivaroxaban versus Warfarin in Nonvalvular Atrial Fibrillation. *The New England Journal of Medicine*.

[5] Granger C. B., Alexander J. H., McMurray J. J. (2011). Apixaban versus warfarin in patients with atrial fibrillation. *The New England Journal of Medicine*.

[6] Giugliano R. P., Ruff C. T., Braunwald E. (2013). Edoxaban versus warfarin in patients with atrial fibrillation. *The New England Journal of Medicine*.

[7] Connolly S. J., Ezekowitz M. D., Yusuf S. (2009). Dabigatran versus warfarin with atrial fibrillation. *The New England Journal of Medicine*.

[8] Levine M. N., Hirsh J., Landefeld S., Raskob G. (1992). Hemorrhagic complications of anticoagulant treatment. *CHEST*.

[9] Chai-Adisaksopha C., Crowther M., Isayama T., Lim W. (2014). The impact of bleeding complications in patients receiving target-specific oral anticoagulants: A systematic review and meta-analysis. *Blood*.

[10] Lao L., Hamilton G. R., Fu J., Berman B. M. (2003). Is acupuncture safe? A systematic review of case reports. Altern Ther Health Med. *Alternative Therapies in Health and Medicine*.

[11] Ernst E., White A. R. (2001). Prospective studies of the safety of acupuncture: a systematic review. *American Journal of Medicine*.

[12] Yamashita H., Tsukayama H., Tanno Y., Nishijo K. (1999). Adverse events in acupuncture and moxibustion treatment: a six-year survey at a national Clinic in Japan. *The Journal of Alternative and Complementary Medicine*.

[13] Melchart D., Weidenhammer W., Streng A. (2004). Prospective Investigation of Adverse Effects of Acupuncture in 97 733 Patients. *JAMA Internal Medicine*.

[14] White A., Hayhoe S., Hart A., Ernst E. (2001). Adverse events following acupuncture: prospective survey of 32 000 consultations with doctors and physiotherapists. *British Medical Journal*.

[15] MacPherson H., Thomas K., Walters S., Fitter M. (2001). The York acupuncture safety study: prospective survey of 34 000 treatments by traditional acupuncturists. *British Medical Journal*.

[16] Park S.-U., Ko C.-N., Bae H.-S. (2009). Short-term reactions to acupuncture treatment and adverse events following acupuncture: a cross-sectional survey of patient reports in Korea. *The Journal of Alternative and Complementary Medicine*.

[17] Mcculloch M., Nachat A., Schwartz J., Casella-Gordon V., Cook J. (2015). Acupuncture safety in patients receiving anticoagulants: a systematic review. *The Permanente Journal*.

[18] Kim Y., Kim S., Cho S. (2014). Safety of acupuncture treatments for patients taking warfarin or antiplatelet medications: Retrospective chart review study. *European Journal of Integrative Medicine*.

